# An assessment of import tariff costs for Italian exporting firms

**DOI:** 10.1007/s40888-020-00202-8

**Published:** 2020-11-18

**Authors:** Ilaria Fusacchia, Alessandro Antimiani, Luca Salvatici

**Affiliations:** 1 Department of Economics, Rossi-Doria Centre for Economic and Social Research, Roma Tre University, Via Silvio D’Amico, 77, 00145 Rome, Italy; 2 European Commission, Brussels, Belgium

**Keywords:** Trade policies, Trade restrictiveness index (TRI), Global trade analysis project (GTAP), Global value chains (GVCs), Value added trade, F13, F17, D58

## Abstract

Since production and trade are increasingly organized within global value chains (GVCs), assessing who effectively pays the cost of protection is not straightforward and since productive processes are internationally fragmented, quantifying the effects of trade policy requires an enhanced analytical framework that takes international input–output linkages into account to assess the implications trade costs have on competitiveness at national and sector levels. This paper defines a new synthetic measure of trade protection based on the value added in trade, capturing the effects that the tariff structure has on exporting firms that rely on imported intermediate inputs. The index, defined in a general equilibrium framework, provides a theoretically sound protection measurement in the context of GVCs. We assess trade protection by computing protection indexes at the bilateral level on both gross imports and imports to exports using the Global Trade Analysis Project computable general equilibrium model. These indexes are used to investigate the relationship between the European Union tariffs and integration of the Italian GVCs. In the case of Italy, imports to exports are overall less protected than gross imports with significant differences at the sector level. Despite the low levels of nominal protection, industrial sectors play a central role in explaining our results. EU tariffs mostly affect Italian exporting firms in the case of chemical products, wearing apparel and leather products.

**Electronic supplementary material:**

The online version of this article (10.1007/s40888-020-00202-8) contains supplementary material, which is available to authorized users.

## Introduction

The long European stagnation following the 2008–2009 global crisis has fuelled renewed debate about the importance of a strong industrial base to sustain and strengthen recovery and foster competitiveness. The debate is going to be even more important in the coming months due to the economic consequences of the COVID-19 epidemic. The design of appropriate trade policy measures to achieve this goal requires a full understanding of the characteristics of the current manufacturing production paradigm in terms of organization of international supply chains and production networks and clear identification of the main linkages between countries and sectors.

About one half of global exchanges is related to global value chains (GVCs). The associated increase in trade in intermediates (that is, parts and components used as inputs in the production of final goods for end consumers) magnifies trade costs that are incurred several times along the chain (Yi 2003). Since the income generation role of exports strongly depends on international exchanges of intermediates and services which are required by domestic firms to produce exported goods, tariffs on imports translate into higher costs associated with a country’s exports. Therefore, restrictive trade policies negatively affect domestic producers' competitiveness in international markets since they reduce access to the most efficient inputs (Cattaneo et al. 2013; Taglioni and Winkler 2014).

The focus of this article is on the challenges posed by the increased complexity of international trade patterns on trade policy analysis. We extend the set of trade restrictiveness indexes proposed initially by Anderson and Neary (2005) to assess the effects of trade policies on GVC-related trade and develop a measure of bilateral trade policy restrictiveness that captures the effects that the tariff structure has on exporting firms that rely on imported intermediate inputs. We use these indexes to investigate how the protection granted by the European Union (EU) tariff structure to the Italian economy affects its integration in global supply networks.

Our framework builds on global input–output accounting and trade in value added (VA). In multi-country production chains, fragments of value added (e.g., the remuneration of factors of production) from different locations are combined to form final goods. Therefore, the empirical assessment of trade policy must acknowledge which country is the source of the value that is embedded in trade. This information can be used to determine who is effectively paying the cost of protection. For instance, firms that require a large share of intermediate imports to export pay higher tax rates in terms of value added (Cusolito et al. 2016).

Standard trade data, recorded on a gross basis, include double counting and do not provide an accurate picture of trade relations. While trade statistics provide the shares of parts and components in gross trade, they do not allow the value to be reallocated to the countries where different stages of production effectively take place (IMF 2013). Global inter-country input–output (ICIO) tables, which put national accounts and bilateral trade data on goods and services into a consistent statistical framework, trace transactions in final and intermediate goods both within and between countries and allow (indirectly) trade to be measured in terms of VA content. This metric allows all backward linkages between countries and sectors to be taken into account and captures the value of the imported inputs used directly and indirectly (at all stages of a country's production) in the manufacturing of exported goods.

From a national account perspective, what is internationally traded is VA and the adequate measure of trade distortion is no longer the nominal tariff structure on the output, but the protection on value added. Relying on such a metric, we can define different benchmarks which can be used to measure restrictiveness according to where the VA originates (domestically or abroad) and how it is used by the importing country. The resulting restrictiveness index is equivalent to the actual trade policies in terms of the impact on the foreign value added embedded in a country's exports.

The empirical analysis is performed using a modified version of the Global Trade Analysis Project (GTAP) model, GTAP-VA (Antimiani et al. 2018a), calibrated to the Version 10 of the GTAP Data Base. Results suggest that the use of the new trade metrics could improve the empirical information used to support policymaking (Koopman et al. 2013). We find that EU tariffs harm Italian producers who use foreign intermediate inputs to produce their exports, especially those sourced from China. Despite the low levels of nominal protection of the EU markets, industrial sectors play a key role in explaining this result: Italian exporters sourcing inputs of chemical products, wearing apparel and leather from abroad are the most affected by the EU tariffs. Our analysis also provides evidence on the differentiated impact the same policy has depending on the structural features of the economy it is applied to. By comparing Italy with Germany, we find that firms in the two countries are affected very differently by the EU Common External Tariff as far as the cost of foreign inputs is concerned. The German firms are overall in a better position than the Italian ones, although there is a substantial variation at the bilateral and sector level.

The paper is structured as follows. In the next section, we discuss the EU trade policy and the methodological challenges in its evaluation and introduce the Italian specialization patterns. Then we present the model and the protection indexes as well as the database used for the empirical application. In Sect. 4, we discuss the results. Section 5 provides some policy implications of our analysis and concludes.

## EU trade policy and Italy's position in GVCs

### EU trade policy and tariff indexes

In this paper, we focus on the most traditional trade barriers—i.e., tariffs—starting with those agreed upon at the multilateral level in compliance with the MFN tariffs that were included in the General Agreement on Tariffs and Trade (GATT)/World Trade Organization (WTO) schedule at the end of the Uruguay Round. Applied rates are generally identical to the WTO bindings and the EU has bound 100% of tariff lines.

Agricultural tariffs stand out from industrial tariffs for several reasons: significantly higher rates, about three-fold; a higher percentage of non-ad valorem rates; and several tariff lines for the implementation of TRQs. On average, bound tariffs on agricultural products remain higher (14.1%) than on non-agricultural products (4.3%) and vary considerably from one agricultural product to another with a standard deviation of 23.7 compared to 4.4 for non-agricultural products. About 25% of tariff lines were duty free in 2014: the sectors with the highest percentages of duty-free lines are for cotton, wood and paper, minerals and metals, and other agricultural products (WTO 2017).

The EU maintains preferential tariffs for imports from certain countries under its reciprocal or preferential agreements. The EU is the largest trading partner for many low- and middle-income countries, and trade preferences make up one of the central policies aimed at improving integration between the EU and these countries. The EU was the first high income importer to introduce preferential policies. Since the 1971 Generalised System of Preferences (GSP), the tide of preferential schemes has continued to rise, significantly widening the number of countries and products covered. Imports of agricultural products from many countries can enter the EU at zero or reduced tariffs under the EU's everything-but-arms initiative, its GSP and GSP + schemes, and its network of trade agreements.

There are two main methodological challenges in the evaluation of any type of trade policy: measurement and aggregation (Cipollina and Salvatici 2008). As far as the former is concerned, measurement of trade policy is perhaps one of the toughest issues faced in the evaluation of trade policy, especially in cases where non-tariff measures are the primary trade policy instrument. However, the problem also arises in the case of non-ad valorem tariffs which constitute about 11% of EU tariff lines and comprise specific, combined, mixed, and other complex forms. As far as aggregation is concerned, even when trade restriction quantification is readily available, as is the case with import tariffs, the information comes at a highly disaggregated level whereas global economic models must aggregate the information to a higher level. The EU's tariff schedule, for instance, includes 9414 tariff lines.

Anderson and Neary (1996, 2005) develop a tariff index theory defined in a general equilibrium framework, taking interdependence between sectors into account, allowing relative prices to adjust and factors to be reallocated across sectors and admitting substitution effects in production and consumption both within and across countries (Ferrarini and Hummels 2014). Their theoretical model provides a consistent aggregation procedure^1^ which solves the endogeneity problem affecting a-theoretical weighting schemes (Cipollina and Salvatici 2008; Anderson et al. 2013; Laborde et al. 2017). These theoretically sound measures provide indexes that are equivalent to the original data in terms of the variable of interest.

Anderson and Neary (1994) assess the effect of the structure of trade policy on national welfare, defining the Trade Restrictiveness Index (TRI) as the uniform tariff that yields the same welfare as the original differentiated tariff structure. Anderson (1998) defines a Distributional Effective Rate of Protection (DERP) as the uniform tariff that yields the same sector specific factor income as the actual tariff structure. This can be used to measure the extent to which the level of protection is translated into sector-specific factor income. Anderson and Neary (2003, 2005) focus on import flows and define the Mercantilist Trade Restrictiveness Index (MTRI) as the uniform tariffs that maintain the value of gross imports at world prices.

Rouzet and Miroudot (2013) compute the 'cumulative tariff' (i.e. the accumulated burden of upstream tariffs for a given importer) which quantifies the total cost-push effect of direct and indirect tariffs, taking into account the upstream GVC structure. Muradov (2017) extends the concept to account for indirect bilateral trade flows and proposes two alternative measures to account for the related costs, the cumulative tariff at origin and destination. Cappariello et al. (2018) use these measures to provide an assessment of the indirect costs of Brexit estimating both the cost-push effect of tariffs and the cumulative resistance of export flows.

Diakantoni et al. (2017) argue that after falling into relative obscurity, at least from a normative perspective, effective protection rates (EPRs) may return to the central stage as international trade moves from "trade in (final) goods" to "trade in tasks". Several contributions (Diakantoni and Escaith 2012; Rouzet and Miroudot 2013; Chen et al. 2016) consider multiple border crossings in the traditional definition of the effective protection rate. More recently, Feenstra (2017) and Diakantoni et al. (2017) extend the concept of effective protection to reflect the impact of import tariffs on the foreign value added in an industry's exports.

However, all these GVC-related measures of tariff indexes are based on simplifying assumptions, e.g., fixed technological coefficients and infinitely elastic supply of factors available to the economy. Consequently, output can instantaneously and costlessly adjust to any variation in the level of final demand. GVCs, on the other hand, are better analysed as a complex set of general equilibrium interdependencies between countries reflecting a combination of preferences, technology, endowments, and policy (Walmsley et al. 2014). Accordingly, Antimiani et al. (2018b), based on the theoretical framework posited by Anderson and Neary (2005), define in general equilibrium different benchmarks which can be used to measure restrictiveness, according to where the value added originates: the resulting Value Added Trade Restrictiveness Indexes are equivalent to the actual trade policies in terms of the impact on domestic or foreign (direct or indirect) value added embedded in imports. In this perspective, our analysis also relates to an increasing number of papers that combine information on tariffs with computable general equilibrium (CGE) models of trade and value added with the aim of investigating the impacts of protectionist measures and trade wars. Freund et al. (2018) use a CGE model to assess the implications of higher bilateral tariffs between China and the US for low- and middle-income countries. In Fusacchia (2019), a simulated multi-region multi-sector general equilibrium model of the global economy is used to evaluate the impacts of tariffs implemented by the US in 2018 and the subsequent retaliation by China. Finally, Bellora and Fontagné (2019) use a general equilibrium framework with intermediate and final products to quantify the effects of detailed tariff changes on value added and welfare.

### Italian specialization patterns

The Italian economy has been characterized by growing integration in international production networks. Its participation in GVCs is nowadays in line with that of Germany, as gauged both by the share of foreign value added embodied in Italian exports and by the share of national value added embodied in partners' exports (Agostino et al. 2016).

The "Made in Italy" sectors, traditional industries such as textiles, wearing apparel and leather products, show a high degree of involvement in GVCs. The Italian traditional pattern of trade specialization with gross data also seems to be confirmed when considering value added exports. However, Italy's trade specialization in almost all sectors of comparative advantage is less prominent considering only the domestic VA content in exports, i.e., the exports net of foreign value added and double counting (Dell'Agostino and Nenci 2018). Having reorganized the production process along with supply networks out of its national borders, the Italian exported manufactured goods contain a significant share of foreign intermediate inputs.

Focusing on manufacturing sectors and using the WIOD 2013 release, Felettigh and Oddo (2016) demonstrate that the negative effect on the dynamics of Italian market shares is due to changes in GVC participation rather than to shifts in its trade specialization. Dell'Agostino (2017) presents an extensive analysis of the composition of the foreign value added in terms of countries that are suppliers of intermediate. In a context of the growing importance of foreign VA in exports, the most striking feature is the increasing role of China and, to a lesser extent Russia, among the main foreign source countries of value added in the Italian exports, and the corresponding decline of EU countries (Germany included). Overall, the results of the most recent studies of Italian trade performance and competitiveness confirm that due to the changing nature of industrialization and trade policy, measures should consider that "no country is an island", but is part of a complex network of competitive and collaborative relationships.

## The empirical model and the data

### The extended GTAP model for value-added analysis and the trade policy indexes

The economic assessment of trade restriction is performed through a modified version of the standard GTAP model, GTAP-VA, a perfectly competitive comparative static global computable general equilibrium (CGE) model incorporating the deconstruction of the gross trade flows to reallocate the value added generated in the production of goods and services back to the countries in which that income is generated (Antimiani et al. 2018a). It is built on general equilibrium theory and designed to assess the inter-regional, economy-wide incidence of economic policies (Hertel and Tsigas 1997). The main advantages of the CGE approach are its solid micro-theoretical underpinning and its economy-wide scope, as well as its detailed inter-sector linkages for each of the economies represented and the complete and consistent coverage of all bilateral trade flows.

The GTAP model underlying our analysis has a symmetric structure, with production and utility functions homogeneous across regions. Utility functions differ by sector, however, and regions differ because the shares of different products in their outputs vary according to local characteristics. The model parameters are mostly drawn from the literature (Hertel 2013). The model assumes the presence of a representative regional household that receives the factor rewards and allocates regional income (through a Cobb–Douglas utility function) between private consumption, government consumption and saving to maximize its utility. The utility function is nested, with a first aggregation made over distinct goods or sectors and in the latter, a choice is made between domestic or imported quantities.

As for the production side, separable, constant returns-to-scale technologies are assumed. A common approach in CGE literature is to model the production side through a sequence of nested constant elasticity of substitution (CES) functions that aims to re-produce the substitution possibilities across the full set of inputs. The firms' conditional demand for components of value added depends on the relative prices of factors of production whereas composite value added and intermediates are used in fixed proportions (a fixed coefficient function of the Leontief type). In the intermediate input side, imported intermediates are assumed to be separable from domestically produced intermediate inputs. However, in the standard GTAP framework, the elasticity of intermediate input substitution is usually set to 0, i.e. no substitution is allowed in the production intermediates mix which becomes a limit for our analysis. Following Antimiani and Cernat (2018), we introduce a further nest for the intermediate bundle, with a positive value for the elasticity of substitution among intermediates (Corong et al. 2017). Based on the assumption used in the Mirage model (https://www.cepii.fr/anglaisgraph/models/mirage.htm), we applied a uniform value of 0.425.

The import demand is modelled following the Armington aggregation structure, with an exogenous differentiation scheme given by the geographical origin of nationally homogeneous products. That is, under Armington trade, the output of each sector is assumed to be a region-specific variety. Consumer and intermediate goods are a CES composite of domestic and trade partner varieties. This specification explains the cross-hauling of similar products and makes it possible to track bilateral trade flows.

The GTAP model is based on a complete IO accounting framework which takes into account all sources and uses of each economic good and all inputs into production. However, it requires some manipulations to perform GVC analysis.

First, in the standard GTAP model, the sourcing of imports occurs at the border, providing information on total purchases of intermediate inputs by firms (domestic and imported), and total purchases of final goods by households, government and for investment (domestic and imported), but not attributing bilateral trade to the consuming agent (e.g., firms or final consumption). This amounts to applying a proportionality assumption which is not realistic. To overcome this limitation of existing models, we consider a richer input–output structure across countries and sectors that we can match with the actual structure reported in input–output tables. We link the import demand for each specific agent to the sourcing country/sector by applying broad economic categories (BEC)-informed shares to bilateral trade (see Sect. 3.2).

Second, value added multipliers are obtained from the cost structure of firms. They combine the sectoral VA shares in each country with the direct and indirect intermediate usage in the productive process. The multipliers are applied to trade which allows the entire value structure underlying gross trade to be retrieved, thus disentangling each country's contribution, in terms of income, in the production of traded goods. This enables us to define the benchmark for the value-added trade restrictiveness index within the GTAP framework. Finally, to compute the uniform tariffs, we define a new variable, *tr*(*r*,*s*), as the product-generic tariff levied on imports from region *r* into region *s*.

**Fig. 1:**
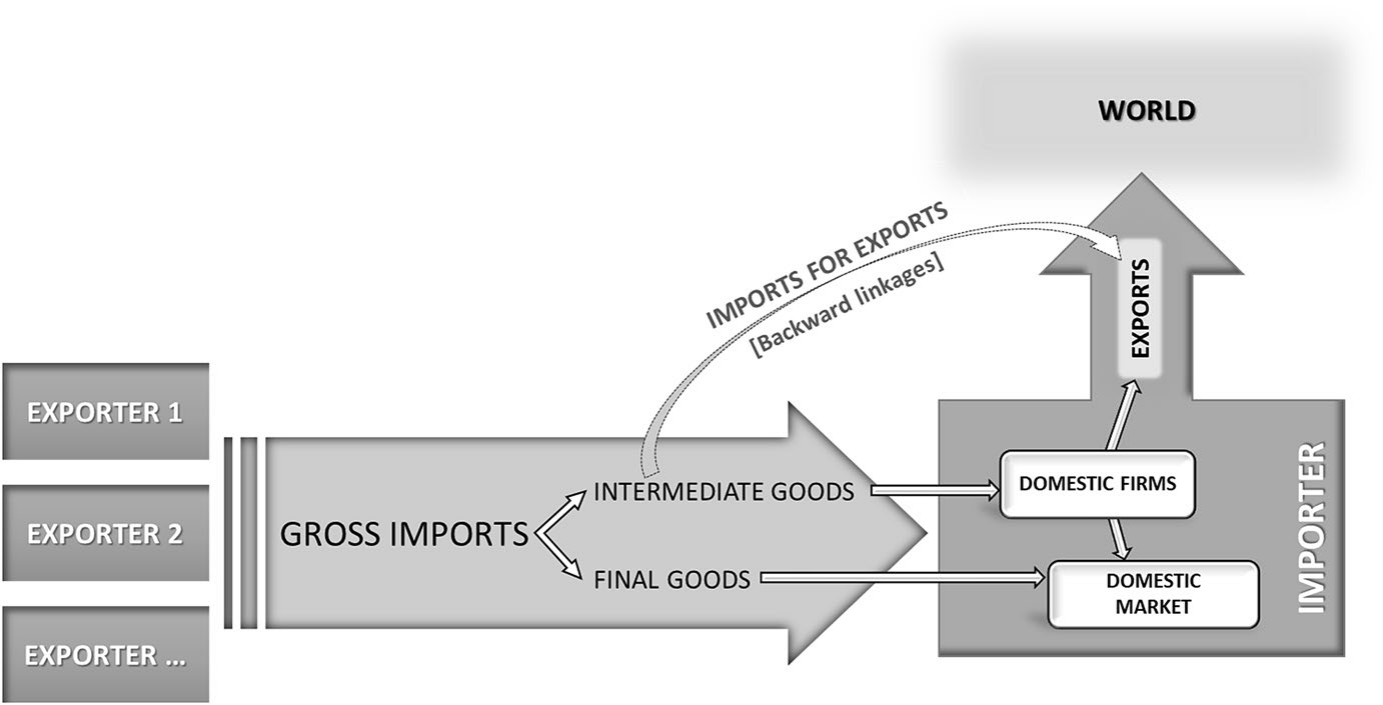
Different types of domestic usage of imports. Source: Authors’ elaborations

We consider trade in intermediate goods and allocate the value added therein contained according to its geographical origin. This enables us to distinguish different portions of value in a country's imports according to the importer's usage of foreign goods (Fig. 1). Specifically, we decompose gross imports into two main components, final goods (directly consumed in the importer's domestic market) and intermediate goods (used as inputs by the importer's domestic firms). Furthermore, in the latter we distinguish between the portion of intermediate inputs used by firms to produce final goods for the domestic market and the portion of intermediates which is embedded in goods exported to foreign markets. This latter category represents the imported content (or the foreign value added) of a country's exports and, as a share of gross exports, provides a measure of the country's backward linkages in GVCs.^2^

In what follows, we specify theory-consistent indexes of trade restrictiveness on both gross and VA bases. First, we provide a decomposition of gross imports based on an input–output framework.

Let *s* and *r* denote countries and i and j sectors. Define $L$ as the matrix of the Leontief coefficients and $V$ as the diagonal matrix with elements equal to the share of direct domestic value added in total output in each sector of each country. The total value-added content of trade flows can be computed using the total value-added multiplier, $VL$, in which the typical element ${v}_{i}^{s}{l}_{ij}^{sr}$ gives the share of country *s*' value added originated in sector *i* of goods produced by country *r* sector *j*. The multiplier matrix provides a breakdown of the flows of value added across country/sector of production since diagonal (off-diagonal) sub-blocks represent domestic (foreign) value added in domestic production. Than define ${F}^{sr}$ as the vector of the final demand for final goods from country s in country r, and ${E}^{r*}$ as the vector of country r's total exports.^3^ Thus, aggregate bilateral imports from a source country s to the importing country r (${M}^{sr}$) can be decomposed in two main components according to the type of domestic usage, e.g., domestic final consumption ($FIN$), either direct (imports of final goods), or indirect (imports of intermediates finally consumed in the importing country), or production for exports ($FVA$): 41

The first term in Eq. (1) gives the value added originated in country s (${V}^{s}$) to produce final goods exported to and directly consumed in country r (without any further processing phase in the importing country). The second term gives the imported value added from country s embedded in the importing country r's production process (${L}^{sr}$) to satisfy domestic final consumption (${F}^{rr}$). The first and the second terms give the value of imports due to final demand in the importing country r. Finally, the third term represents the foreign value added (from country s) in country r's imports which is embedded in the production of its exports ${E}^{r*}$.

The last component can be characterized as GVC-related trade since it includes goods and services crossing more than one border, thus involving at least two production stages located in different countries before the final product reaches the destination market (Borin and Mancini 2017). We used this metric as the VA benchmark used to measure trade policies.

First, we define an index of tariffs which equals the uniform tariff that yields a constant volume of bilateral gross imports as: 5

2 $$ MTRI^{sr} :M^{sr} \left[ {\left( {1 + \tau^{\left( \mu \right)sr} } \right)p^{I} \left( T \right),b^{0} ,\omega } \right] = M^{sr} \left[ {p^{0} ,p^{I} \left( T \right),b^{0} ,\omega } \right] . $$

Next, we define our GVC-related index as the uniform tariff that, if imposed on imports instead of the existing structure of protection, would leave the importer's foreign value added in exports at its current level. Following the definition provided in Eq. 1, it is given by:

3 $$ FVATRI^{sr} :FVA^{sr} \left[ {\left( {1 + \tau_{j}^{\left( \mu \right)sr} } \right)p^{I} \left( T \right),b^{0} ,\omega } \right] = FVA^{sr} \left[ {p^{0} ,p^{I} \left( T \right),b^{0} ,\omega } \right] . $$

In Eqs. (2) and (3), superscript $0$ refers to the reference period so that $b^{0}$ expresses the equilibrium at the point of reference which has to be maintained once the uniform tariff replaces the initial tariff structure and $p^{0}$ are the initial prices. International prices ($p^{I}$) are expressed as a function of the tariff vector ($T$) to allow for endogenous world prices thus dropping the small country assumption (Salvatici, 2001; Antimiani and Salvatici, 2005). The right-hand side in both equations is, respectively, the total value of imports and the foreign value used to export embedded in bilateral imports at the initial non-uniform tariffs. The left-hand side maintains the same values when applying a uniform (product-generic) tariff ($\tau^{\left( \mu \right)} )$.

### The extended GTAP Data Base

In this study, data are taken from version 10 of the GTAP Data Base for a baseline of consistent data on consumption, production and trade (Aguiar et al. 2019). The GTAP Data Base is a fully documented global database that provides comprehensive and balanced data on production, bilateral trade, transport and trade policies, covering 121 countries (representing 98% of world GDP and 92% of the world population) and 20 aggregate regions for 65 commodities.

The advantage of using the GTAP Data Base for a trade in value added analysis is that it reconciles data from different sources and puts them into one consistent database with a broad sectoral and regional coverage. However, to implement the FVATRI, a four-dimensional information level on the source and destination country-sector is required. At the same time, the database itself does not account for how imported intermediate products are used. Within the GTAP framework, imports of intermediates from all countries are aggregated at the product level at the border into a composite imported good. This composite good is then allocated across sectors and uses based on relative demands and shares. Using this approach, we cannot trace exports of intermediates from one country into the production processes of another, and following on from that, into their contributions to other countries' exports. Furthermore, we cannot directly identify the industry-to-industry trade required for the construction of ICIO data, neither can we link trade flows directly from producers in each region to importing firms and consumers in all other regions, which is required to implement the above imports decomposition. Different methods exist in which supplementary information is used to distinguish between countries of origin on an industry-use basis. A commonly used approach is to apply proportionality, e.g., using the shares of imports used by firms on the total country's imports and applying them to bilateral trade. The key problem with this method is that it ignores differences in the types of imports from different regions. For a given product, some countries’ exports may target final demand whereas others may target intermediate demand. In this analysis, we apply a more refined method using a series of concordances from the United Nations Statistics Division (UNSD)^6^ to obtain BEC-informed shares that are needed to attribute bilateral imports in the GTAP Data Base at the agent level (i.e., firms, government, private households).

Specifically, we start with UN COMTRADE import data at the six-digit level of the Harmonized Commodity and Coding System (HS) and apply the first concordance between HS and the BEC Rev.5. Each economic category is completely decomposable by end use. Accordingly, the mapping between BEC and the System of National Accounts (SNA) end-use dimension makes it possible to identify three different end use classes, namely, intermediate consumption, gross fixed capital formation and final consumption. Finally, the HS-GTAP concordance is applied to map each HS line to a GTAP commodity which gives the BEC-informed shares. A similar procedure is applied by Aguiar et al. (2016), Liapis and Tsigas (2014), and Walmsley et al. (2014).

Protection data in the GTAP Data Base are sourced from the Market Access Maps (MAcMap).^7^ It provides a set of consistent and exhaustive *ad valorem* equivalents of applied border protection worldwide. However, one caveat is that it does not include information about tariff exemptions granted in export processing zones and through inward and outward processing trade regimes. These regimes introduce a differential tariff treatment of imports depending on the sectors and the firms to which they are destined since imported goods that enter into the production of exports are not subject to import duties. They are particularly relevant in the case of China trade flows (Yu and Tian 2012).

We aggregate the GTAP Data Base in 12 countries and regions, identified in terms of their relevance as Italy's suppliers of goods and services imports in 2014, the benchmark of the dataset. Together, the extra-EU countries considered in our aggregation account for more than 50% of extra-EU Italy's imports. In the discussion of our findings, we do not present results for Russia (because of the extreme concentration of the extractive sector) or for Switzerland and Turkey (due to the extremely low level of tariffs they face in the EU). The sectoral aggregation consists of 30 sectors and keeps all manufacturing sectors disaggregated. Details on the aggregation are reported in Table 1.

**Table 1:** GTAP Data Base aggregation

**Countries and regions**	
Italy	Switzerland
Germany	India
Rest of EU28	Turkey
China	Japan
Russia	Brazil
US	Rest of the World
**Commodities and activities**	**GTAP code**
Primary sectors	pdr, wht, gro, v_f, osd, c_b, pfb, ocr, ctl, oap, rmk, wol
Forestry and fishing	frs, fsh
Mineral extraction	coa, oil, gas, oxt
Meat sector	cmt, omt
Vegetable oils	vol
Dairy	mil
Rice	pcr
Sugar	sgr
Other processed food	ofd
Beverage and tobacco	b_t
Textile	tex
Wearing	wap
Leather	lea
Wood sector	lum
Paper and publishing	ppp
Oil products	p_c
Chemicals	chm
Pharmaceuticals	bph
Plastic products	rpp
Non-metallic products	nmm
Iron and steel	i_s
Non-ferrous metals	nfm
Metal products	fmp
Computer and electronic	ele
Electrical equipment	eeq
Machinery and equipment	ome
Motor vehicles	mvh
Other transport equipment	otn
Other manufacturing	omf
Services	ely, gdt, wtr, cns, trd, afs, otp, wtp, atp, whs, cmn, ofi, ins, rsa, obs, ros, osg, edu, hht, dwe

**Fig. 2:**
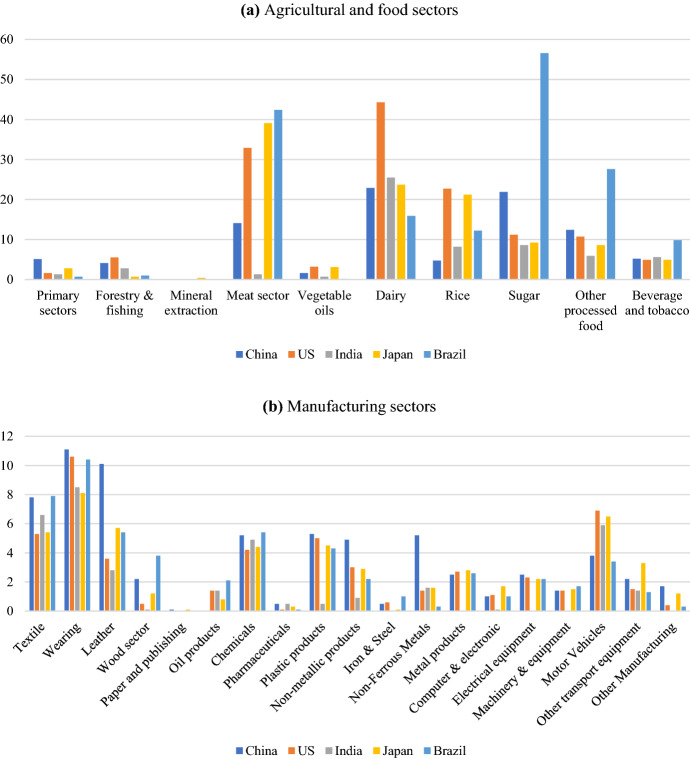
EU's import tariffs, selected trade partners (% ad valorem rates, 2014). Source: GTAP 10 Data Base

Figure 2 records the tariff rates applied by the EU to the trade partner considered in our analysis.

EU tariffs are more relevant for the agricultural and food sectors. For these sectors, tariffs can be higher than 50%, as for sugar. On the contrary, manufacturing sectors tariffs are more homogeneous and show lower peaks given that the highest rates never reach 12%.

## Results

### Gross imports and imports for exports

In this section, we review Italian imports and firms' international linkages, with some descriptive statistics on sectoral backward integration and patterns of imports both on a gross and a value-added basis with selected trade partners.

**Fig. 3:**
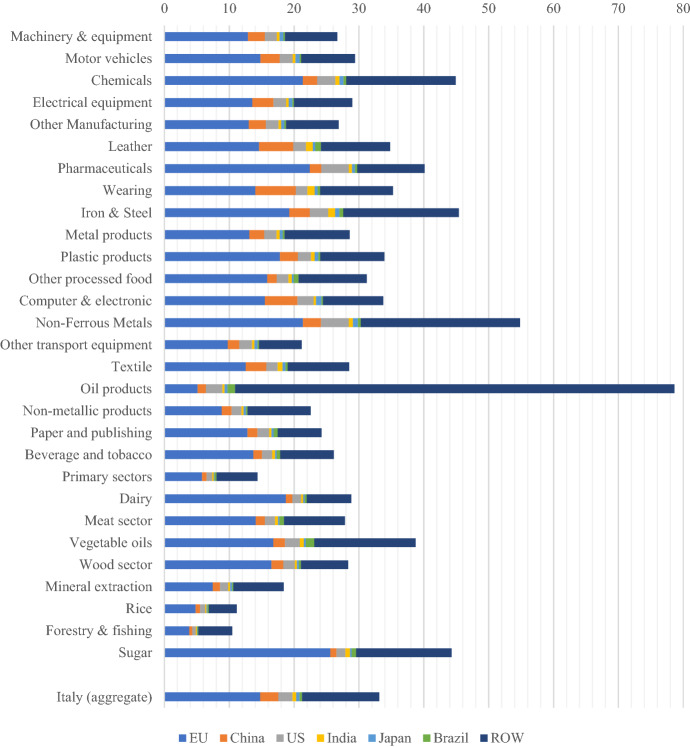
Italy's backward integration and FVA share on total exports by sector (2014). Source: Authors’ computation using the GTAP-VA model

Figure 3 shows the aggregate backward integration for Italy (i.e., the use of foreign inputs as a share of gross exports) and the use of foreign intermediate inputs used by Italian firms to exports (i.e., the sourcing of foreign inputs to export by each Italian sector as a share of gross sectoral exports). Sectors are ordered according to their importance in gross exports.

Overall, Italy appears to be significantly integrated into international production networks as a buyer of intermediates used in its exports, showing an aggregate backward integration index value in 2014 of 33.1% (18.4% for extra-EU imports). These figures are slightly lower than the German ones (35.9 and 19.2%, respectively) and broadly in line with the other EU countries. It is worth recalling that getting embedded in global value chains is a powerful determinant of export growth. It has a positive effect on the domestic value added (e.g., remuneration of domestic factors of production) even if it implies that a growing share of gross exports represents the value added that has been produced in foreign countries (Altomonte et al. 2018).

There is significant heterogeneity in terms of backward integration by sector. The sectors based on natural resources show the highest levels of dependency on imported inputs as in the case of oil products (about 80%) and non-ferrous metals (more than 50%). Chemicals, an important exporting sector for Italy, embeds about 45% of foreign value added in its exports, mainly sourced regionally from other EU economies. The other EU countries are significant providers and in most cases, they provide more than 50% of total foreign inputs. Among the extra-EU providers, the role of China is apparent but overall, these figures confirm the importance of regional value chains in the development of GVCs (Baldwin and Lopez-Gonzalez 2015). Indeed, the large EU share shows that Italy is part of the dense European intermediate goods network known as 'Factory Europe'.

**Fig. 4:**
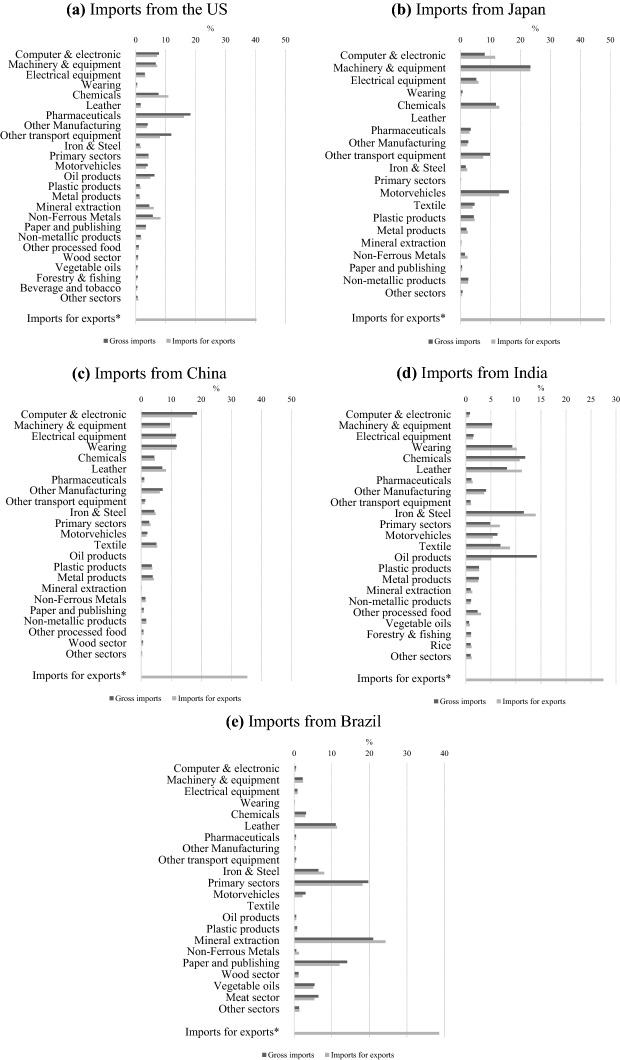
Italy's sector shares for gross and intermediate imports, and total intermediate imports share (selected partners, 2014). *Share on total imports. Source: Authors’ computation using the GTAP-VA model

Next, we focus on Italy's import structure from the most important exporters: US, Japan, China, India and Brazil. In Fig. 4, we compare sector shares for *gross imports* with the shares for foreign value added embedded in Italian exports by each foreign exporting sector (*imports for exports*). At the bottom of each panel, we report the ratio between total intermediate and imports.

On average, around 38% of Italy's imports from the five countries considered represents intermediate inputs used by Italian firms to produce their exports, a share that is slightly higher than the overall average (Fig. 3). There are huge variations among exporters: the largest share is registered by Japan (48%), and the lowest by India (27%), suggesting that GVC integration is deep(er) between high-income countries.

Sectoral concentration, as well as sectoral composition, is quite similar since the largest import flows are relevant both for the domestic market and export production. However, gross imports seem to be more concentrated than imports for exports in all cases except for Brazil.

The share of iron and steel foreign value added is always larger since this sector provides crucial inputs for Italian exports. On the other hand, the gross import share is always larger for transport equipment and motor vehicles suggesting that they are mainly used for final consumption in the domestic market.

For the other sectors, the patterns are quite differentiated and exporter-specific. Computer and electronics imports from Japan and wearing apparel and leather from India are more important as inputs for exports. In contrast, pharmaceuticals from the US and oil products from India are mostly imported for domestic consumption.

### The protection on gross and value-added trade

Given the complex nature of (value added) trade flows, the evaluation of the impact of trade policies requires standard (gross) trade statistics to be complemented with trade metrics on a value-added base in order to take into account the backward linkages. Accordingly, we assess the impact the EU trade policy has on Italian imports not only in terms of total flows through the MTRI, but also in terms of intermediates used by firms to exports through the FVATRI. This is done on a bilateral basis for the leading exporters. To quantify the protection granted by the EU tariffs to the Italian economy, we keep constant extra-regional imports (e.g., excluding intra-EU flows) in both gross and VA terms. Uniform tariff equivalents are then obtained by setting bilateral tariffs to zero and replacing them with the uniform one that keeps constant imports either in gross value or in value added. The trade flows considered only include goods since services are not subject to any tariffs.^8^

Table 2 reports the uniform tariff equivalents on total Italian gross imports (MTRI) and foreign value added in exports (FVATRI) as well as the corresponding values for the main trading partners.

**Table 2:** Uniform tariff equivalent rates, Italy's imports

	MTRI	FVATRI
Total imports	4.2	2.0
Exporter		
US	2.2	1.7
Japan	3.6	2.7
China	4.1	3.2
India	3.2	1.8
Brazil	13.9	1.8

Source: Authors’ simulations using the GTAP-VA model

The average MTRI value is equal to 4.2% and there are large differences across exporters. Such differences are not surprising given the discrepancies between the trade-weighted averages presented in Fig. 2. However, looking at the differentiation among exporters, it is worth noting that the protection ranking is certainly not consistent with the expectations in terms of preferential access or bilateral trade agreements. Indeed, the lowest tariff barriers (2.2%) are faced by the US, a high-income country which is not part of any bilateral agreement.

The FVATRI values are always lower than the MTRI ones, and the smaller range of variation could be expected given that the value of intermediate goods cannot exceed the gross value. The average FVATRI (2%) is less than half of the MTRI, but the difference between the two indexes varies a great deal across exporters: the tax on intermediate goods is close to overall protection in the case of China, US and Japan whereas it represents a small percentage in the case of Brazil. It is worth noting the change in the exporter ranking where China is the country facing on average the highest protection on the exported value added (3.2%).

**Fig. 5:**
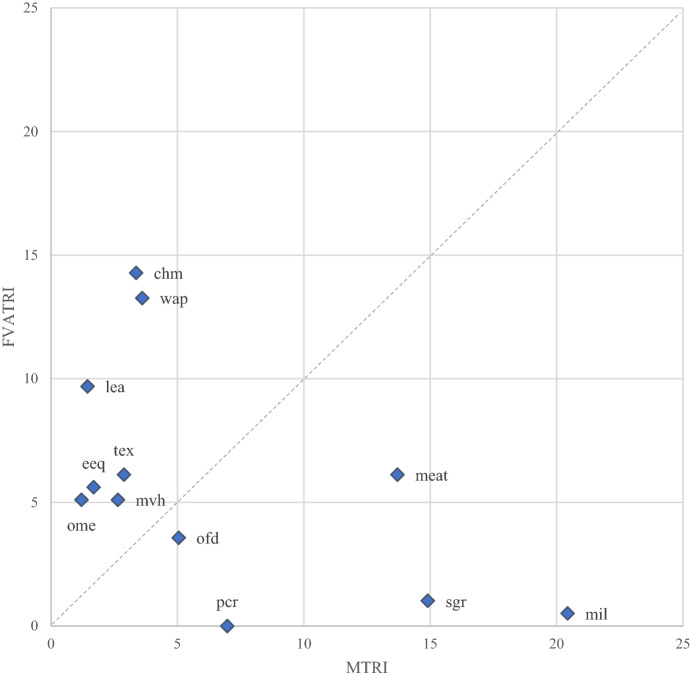
Sector shares on the trade restrictiveness indexes for Italy (2014). Blue dots represent sectors’ weights (percentage of the total index). Only sectors with a weight above 5% for at least one of the two indexes are presented. Source: Authors’ simulations using the GTAP-VA model

To explain what drives these results, we compute the weight of each sector on the indexes level. Figure 5 shows the sectoral shares on the trade restrictiveness indexes for Italy, computed with the two metrics of gross and VA flows. For the readability of the graph, only the most relevant sectors are reported.

The vertical axes of the diagrams display the weight each sector has on the FVATRI expressed as a percentage of the total of the index. On the horizontal axes, sectoral weights are displayed for the MTRI. Then, the sectors above the bisector are those whose tariffs are more relevant in the production of Italian exports. Similarly, the sectors which are more relevant for the protection in gross terms are below the bisector.

The importance of sectors is different when considering gross or VA imports. Although industrial sectors overall face low levels of nominal protection in the EU markets (Fig. 2), they show a prominent role in explaining the FVATRI as in the case of Chemical products, Wearing apparel or Leather products. The opposite is true in the case of agrifood sectors such as Dairy, Sugar or Meat. Finally, there are sectors such as Motor vehicles or Food products where tariffs affect both exporting firms and domestic consumers.

Given that these sectors play quite a different role in bilateral export flows, this sheds some light on the bilateral protection scores previously mentioned. For instance, Meat plays a prominent role in Brazilian exports whereas Wearing apparel and Leather products are quite significant in Chinese exports.^9^

### Common tariffs but different protection levels: a comparison with Germany

In this section, we show that the same trade policy, namely the Common External Tariff of the EU, leads to different outcomes according to the structural features of the economy it is applied to. To this end, we compute the protection indexes for the largest EU economy, i.e. Germany. Comparing Italy with Germany seems a reasonable choice given their levels of integration in global and regional value chains. We have already mentioned that backward participation indexes are similar for both countries and they also get similar shares of FVA from EU partners: 46% for Germany, 44% for Italy.

On the other hand, Italy and Germany present different trade specialization patterns (Accetturo and Giunta 2016). Accordingly, a comparison of the tariff equivalents between the two countries allows us to show how the impact of a trade policy depends not only on the tariff structure (which is by definition a common feature in a customs union), but also on the structure of the economic system to which it is applied and, in the case of the FVATRI, on the pattern of integration in global networks.

The comparison between Tables 2 and 3 is quite striking. The German MTRI (5.3%) is higher than the Italian one while the opposite is true for the FVATRI. Accordingly, German consumers are more negatively affected than the Italian ones while German firms are in a better position than the Italian ones.

**Table 3:** Uniform tariff equivalent rates, Germany's imports

	MTRI	FVATRI
Total imports	5.3	1.7
Exporter		
US	2.8	2.0
Japan	2.1	2.1
China	3.4	2.6
India	3.7	1.5
Brazil	11.3	1.3

Source: Authors’ simulations using the GTAP-VA model

The comparison also shows significant differences at the bilateral level. Chinese, Indian and Brazilian gross exports are less taxed in the German market whereas US intermediate exports are less taxed in the Italian market. It is also worth mentioning that the ratio between FVATRI and MTRI is roughly similar for both countries but in the case of Japan since the two indexes coincide for Germany.

**Fig. 6:**
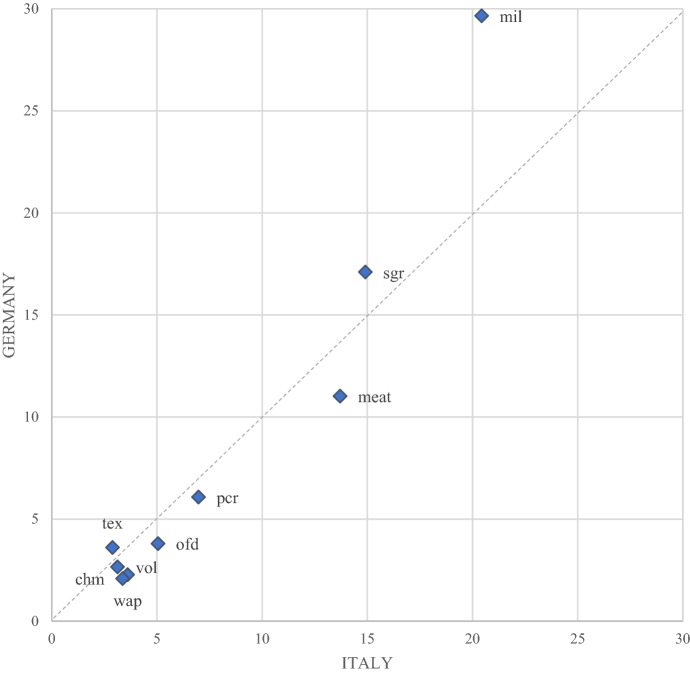
MTRI sector decomposition for Italy and Germany (2014). Blue dots represent sectors’ weights (percentage of the total index). Only sectors with a weight above 3% for at least one of the two indexes are presented. Source: Authors’ simulations using the GTAP-VA model

To shed some light on the role of different sectors, Fig. 6 combines the MTRI sector decomposition for Italy and Germany. The horizontal axes of the diagrams display the weight each sector expressed as a percentage on the total of the Italian index. On the vertical axes, sectoral weights are displayed for the German MTRI and the sectors below the bisector are those whose tariffs are more relevant in the Italian final goods market. Similarly, the sectors which are more relevant for the protection of the final goods in the German market are above the bisector.

As expected, given the tariff levels (see Fig. 2), agrifood sectors are the most protected in both markets. Dairy products are more protected in Germany and Meat products in Italy but, overall, the most relevant sectors reported in the graph lie near the bisector. According to the sector weights, the same products turn out to be the most (or least) protected ones in both markets.

**Fig. 7:**
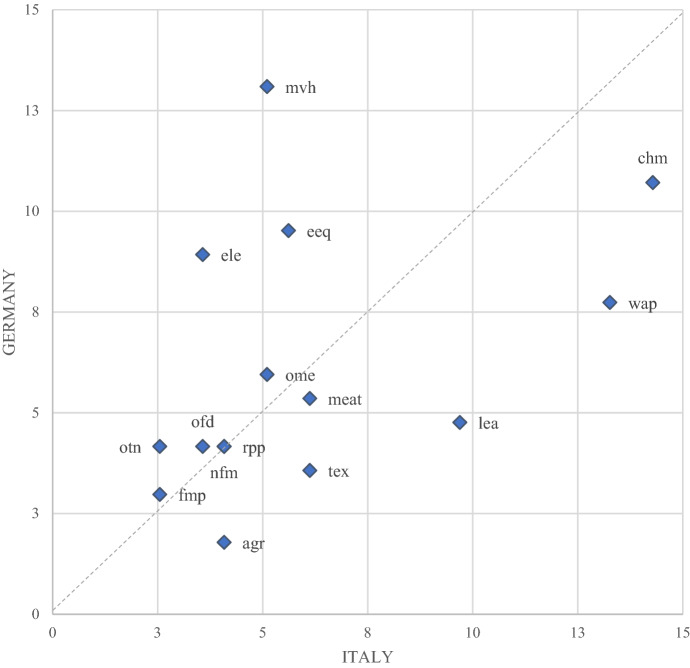
FVATRI sector decomposition for Italy and Germany (2014). Blue dots represent sectors’ weights (percentage of the total index). Only sectors with a weight above 5% for at least one of the two indexes are presented. Source: Authors’ simulations using the GTAP-VA model

The picture is quite different when we perform the same decomposition for the FVATRI (Fig. 7).

Two main features emerge from the graph. First, the weight of the sector is not directly related to nominal tariffs since the demand for foreign intermediate goods depends not only on their prices but also on the prices of the goods using them as inputs. Second, there is not a clear correlation between Italian and German weights. The most negatively affected sectors are Motor vehicles, Electrical equipment and Computers in Germany and Chemicals, Wearing apparel and Leather products in Italy.

The EU Common External Tariff provides similar levels of protection (within sectors) to domestic produces in Germany and Italy. However, German and Italian firms are affected very differently by the EU tariffs as far as the cost of foreign inputs is concerned. Accordingly, we can expect the Italian and German governments to have different priorities in terms of trade negotiation both in terms of countries and sectors to be targeted.

## Policy implications and conclusions

Although the quantification of the impact of trade policy on prices, economic activity and welfare have always been at the core of trade policy analysis, the complexity of today’s trade relations raises new unprecedented challenges. In particular, the rise of GVCs requires the adoption of enhanced analytical frameworks that take the international input–output linkages into account. Since exports rely on imported inputs, the evaluation of trade policies requires the use of new trade metrics based on the value-added components to assess the implications of trade costs on competitiveness at national and sector levels. In this paper, we define a new protection index framework and show how it can be operationalized for quantitative trade policy analysis.

We then apply the proposed index to the quantitative analysis of the EU tariffs affecting Italian imports. The index is computed through an applied general equilibrium model of the global economy with trade in both final and intermediate products. By mapping bilateral supply chain linkages and value-added flows, the model provides a rich framework that captures countries’ heterogeneity in terms of the composition of their trade flows as well as in terms of their involvement in GVCs.

The main caveat is that the model is based on comparative statistics and a few crucial assumptions, for instance, in terms of employment levels or international labour mobility. Consequently, the model’s results do not include dynamic effects such as the effects on productivity and growth, unemployment and migration. On the other hand, the simulation model allows the impact of trade policies to be explored through the reorganizations of the GVCs and shows that some tariffs impacts differ across countries depending on the sectoral composition of their economies and the relative importance of different foreign markets. Properly accounting for observed GVC linkages makes a substantial difference to the quantification of the general equilibrium effects of protectionist trade policies. In this respect, the FVATRI represents a useful addition to the tools available for policy analysis since it takes into account how cross-border multi-stage production affects the transmission of trade policy to national welfare.

This study provides an assessment of the protectionist impact of EU tariffs, with a focus on Italy. The main policy implication of our analysis is that bilateral nominal tariffs and trade flows alone do not provide an accurate picture of the impact of protectionist measures through backward and forward linkages.

The value of the index for foreign value added in the exports component is indicative of the harm inflicted to domestic producers using foreign intermediate inputs to produce their exports. This shows the ‘*beggar thyself*’ content of protectionism. By exploiting the methodology for value-added accounting of trade flows, we find that the EU tariffs impose a burden of up to around 3.2% in the case of Italian imports from China. The figure is not trivial considering that it is an average and taking tariff rates variability into account.

Indeed, manufacturing sectors imports play a significant role in explaining the protection of imports for exports, notwithstanding the low levels of nominal EU tariffs. In particular, we find that EU tariffs mostly affect Italian exporters sourcing inputs of chemical products, wearing apparel and leather from abroad.

We also document a significant difference in the impact of the EU tariffs on Italy and Germany. Since the nominal tariffs are (by definition) the same, this highlights how the same policy has a different impact according to the country in which it is implemented. The German market is more protected, but Italian firms pay a higher cost for the foreign inputs they need to export.

Finally, our results shed some light on the implications of the most recent trade policy moves of the EU Commission. Italian consumers and firms are going to benefit more than German ones from the latest bilateral agreements with Japan and (possibly) Brazil. On the other hand, German consumers and firms are already more negatively affected when they import from the US and the situation could deteriorate if there are escalating transatlantic trade wars.

## Electronic supplementary material

Below is the link to the electronic supplementary material.

Supplementary file1 (DOCX 28 kb)

## Footnotes

1 In the presence of very differentiated tariff structures (which is the rule), aggregation is required for policy purposes since the information on production and demand structures needed to make a full assessment of the implications of trade policy is available at a much higher level of aggregation than the information on tariffs and trade flows.

2 Conceptually, the foreign value added in exports (FVA) term recall the “import to export” (I2E) measure proposed by Baldwin and Lopez-Gonzales (2015). However, I2E is a recursive metric with pervasive double counting. The trade in VA approach used in our analysis overcomes this difficulty.

3 The star represents the sum of all destination countries except r.

4 For ease of exposition, in Eq. (1) we are assuming that the exporting country *s* uses only domestic inputs to export both final and intermediate goods to country *r*. This assumption is relaxed in the empirical application provided in Sect. 4. A detailed derivation is provided in Fusacchia (2017).

5 The formal derivation of the index is provided in Anderson and Neary (2003).

6 Correspondence tables are retrieved from: https://unstats.un.org/unsd/classifications/econ/.

7 For the documentation, see Guimbard et al. 2012.

8 The experiments imply shocking the tariff for a single member of the EU (e.g., Italy), and this flies in the face of the rules of any custom union. However, it is worth recalling that our simulations are not meant to be realistic: the sole purpose is the counterfactual computation of the protection indexes.

9 Sectoral weights on the indexes at the bilateral level are provided in the Appendix.
