# Supplementary material for: An assessment of import tariff costs for Italian exporting firms
Source: Econ Polit (Bologna). 2020 Nov 18;38(1):31–56. doi: 10.1007/s40888-020-00202-8 (PMC7671830; doi:10.1007/s40888-020-00202-8)
Supplement: Supplementary file 1 — Supplementary file1 (DOCX 28 kb) [file 40888_2020_202_MOESM1_ESM.docx]

**Appendix**

**Table A1**. Sector share on uniform tariff equivalents for Italy's imports (main exporters, % on total rate)

| *Exporter:* | *US* | | *Japan* | | *China* | | *India* | | *Brazil* | |
| --- | --- | --- | --- | --- | --- | --- | --- | --- | --- | --- |
|  | mtri | fvatri | mtri | fvatri | mtri | fvatri | mtri | fvatri | mtri | fvatri |
| agr | 5.0 | 4.6 | 0.3 | 0.8 | 1.2 | 6.6 | 0.6 | 4.6 | 0.1 | 1.1 |
| meat | 19.5 | 6.3 | 0.0 | 0.4 | 0.0 | 0.3 | 0.0 | 0.0 | 90.3 | 24.0 |
| sgr | 0.0 | 0.6 | 0.0 | 0.0 | 0.0 | 0.0 | 0.3 | 0.0 | 3.2 | 6.7 |
| ofd | 3.6 | 4.6 | 0.3 | 0.8 | 1.4 | 1.3 | 1.9 | 0.0 | 0.1 | 3.4 |
| tex | 1.4 | 4.6 | 8.8 | 7.1 | 9.4 | 15.8 | 17.0 | 23.4 | 0.0 | 3.4 |
| wap | 3.6 | 1.1 | 1.6 | 0.8 | 32.4 | 8.5 | 29.6 | 17.7 | 0.1 | 0.6 |
| lea | 1.8 | 1.1 | 0.0 | 0.0 | 16.4 | 11.7 | 12.3 | 16.6 | 2.7 | 16.2 |
| p_c | 1.8 | 0.0 | 0.0 | 1.1 | 0.0 | 0.0 | 3.8 | 1.7 | 0.1 | 0.0 |
| chm | 13.1 | 25.9 | 12.9 | 20.3 | 5.8 | 13.3 | 19.8 | 22.3 | 1.0 | 16.8 |
| rpp | 3.2 | 10.3 | 5.8 | 10.2 | 4.1 | 6.6 | 0.9 | 1.1 | 0.2 | 6.7 |
| nmm | 1.4 | 2.3 | 1.6 | 3.0 | 1.7 | 4.7 | 0.3 | 0.6 | 0.0 | 0.6 |
| i_s | 0.0 | 0.6 | 0.0 | 0.4 | 0.2 | 0.9 | 0.0 | 0.0 | 0.3 | 3.4 |
| nfm | 4.1 | 6.3 | 1.6 | 9.8 | 2.2 | 12.0 | 0.3 | 2.3 | 0.1 | 2.8 |
| fmp | 1.8 | 6.3 | 1.4 | 4.5 | 2.4 | 2.8 | 0.0 | 0.0 | 0.0 | 3.9 |
| ele | 5.4 | 3.4 | 3.8 | 9.0 | 3.9 | 3.5 | 0.0 | 0.0 | 0.1 | 1.1 |
| eeq | 4.1 | 4.0 | 3.3 | 4.9 | 7.2 | 4.1 | 0.0 | 0.0 | 0.1 | 1.1 |
| ome | 5.4 | 5.7 | 11.3 | 10.2 | 4.8 | 3.5 | 0.0 | 0.0 | 0.2 | 1.7 |
| mvh | 10.0 | 3.4 | 30.5 | 11.3 | 1.7 | 0.9 | 8.5 | 2.3 | 0.6 | 1.1 |
| otn | 10.0 | 4.0 | 14.6 | 3.4 | 1.2 | 0.3 | 1.3 | 1.7 | 0.1 | 0.0 |
| omf | 1.4 | 1.7 | 1.9 | 1.5 | 3.4 | 1.3 | 0.0 | 0.0 | 0.0 | 0.0 |

*Note*: sectors with a weight lower that 3% are not included.

*Source*: Authors’ simulations using the GTAP-VA model.
